# Prophages in Skin Pathogens: From Virulence to Therapy

**DOI:** 10.3390/pathogens15060599

**Published:** 2026-06-02

**Authors:** Abirami Karthikeyan, Aqib Javaid, Grace Naa Ayorkor Charway, Nazia Tabassum, Tae-Hee Kim, Young-Mog Kim, Won-Kyo Jung, Fazlurrahman Khan

**Affiliations:** 1 Industry 4.0 Convergence Bionics Engineering, Pukyong National University, Busan 48513, Republic of Korea; abirami@pukyong.ac.kr; 2 Interdisciplinary Program of Marine and Fisheries Sciences and Convergent Technology, Pukyong National University, Busan 48513, Republic of Korea; aqibj@pukyong.ac.kr (A.J.); grace@pukyong.ac.kr (G.N.A.C.); 3 Research Center for Marine Integrated Bionics Technology, Pukyong National University, Busan 48513, Republic of Korea; nazia99@pukyong.ac.kr (N.T.); taehee@pknu.ac.kr (T.-H.K.); ymkim@pknu.ac.kr (Y.-M.K.); 4 Marine Integrated Biomedical Technology Center, The National Key Research Institutes in Universities, Pukyong National University, Busan 48513, Republic of Korea; 5 Ocean and Fisheries Development International Cooperation Institute, Pukyong National University, Busan 48513, Republic of Korea; 6 Department of Food Science and Technology, Pukyong National University, Busan 48513, Republic of Korea; 7 Major of Biomedical Engineering, Division of Smart Healthcare, College of Information Technology and Convergence and New-Senior Healthcare Innovation Center (BK21 Plus), Pukyong National University, Busan 48513, Republic of Korea; 8 International Graduate Program of Fisheries Science, Pukyong National University, Busan 48513, Republic of Korea

**Keywords:** prophages, skin pathogens, treatment, antibiofilm, antivirulence

## Abstract

Prophages are bacteriophage genomes that are part of bacterial chromosomes. They are not just dormant passengers; they actively shape pathogen biology. For example, in skin-infecting pathogens such as *Staphylococcus aureus*, *Streptococcus pyogenes*, and *Pseudomonas aeruginosa*, prophages carry important virulence factors, cytotoxins, superantigens, immune evasion clusters, and epigenetic regulators that directly affect the course of skin and soft tissue infections. This same prophage biology provides a therapeutic strategy: prophage-derived molecules, including endolysins, holins, spanins, and polysaccharide depolymerases, demonstrate potent antimicrobial and antibiofilm activity against drug-resistant skin pathogens, with several candidates now in clinical development. Engineered chimeric lysins, CRISPR-encoded prophage delivery systems, and the systematic mining of the skin microbiome phageome collectively enhance the translational potential of this biology. This review integrates mechanistic insights into prophage-mediated virulence. It assesses the translational landscape of prophage-derived therapeutics, delineating the conceptual and clinical frontiers that characterize the forthcoming chapter in this domain.

## 1. Introduction

Bacteriophages infect bacteria through two distinct life cycles. In the lytic cycle, phages immediately reproduce and lyse the host cell. Alternatively, the lysogenic cycle entails the integration of phage DNA into the bacterial chromosome as a prophage, in which it remains dormant and is passively replicated with the host genome [1,2]. Lysogenic bacteriophages can switch from dormancy to lytic activation in response to environmental stressors, including antibiotic exposure, UV radiation, or nutrient limitation [3]. Biofilms are self-produced extracellular matrices of DNA, proteins, lipids, and polysaccharides, and this structure enhances bacterial survival by reducing antimicrobial penetration and increasing tolerance to environmental stress [4]. Biofilm-associated bacteria showed 100-fold resistance to antibiotics and host immune defense compared to planktonic cells, posing a major challenge for the treatment of chronic infections [5]. The global healthcare cost of biofilm-associated chronic infections is estimated at $386.8 billion. In the United States, chronic conditions affect approximately 50% of the population. *Staphylococcus aureus*, *Streptococcus pyogenes*, and *Pseudomonas aeruginosa* delayed wound healing and increased mortality [6,7]. Conventional antimicrobial treatment is often ineffective against biofilm-associated infections because of drug penetration, underscoring the urgent need for alternative therapeutic approaches [8]. Skin and soft tissue infections impose a substantial global health burden, accounting for millions of hospitalizations annually and an accelerating share of antibiotic treatment failures driven by multidrug-resistant (MDR) pathogens [9]. While the virulence of the principal skin pathogens *S. aureus*, *S. pyogenes*, and *P. aeruginosa* has long been attributed to chromosomally encoded factors, prophages are now recognized as central contributors to their pathogenic identities [10]. Prophages, the integrated genomic residues of temperate bacteriophages, are pervasive in the genomes of skin pathogens. Most *S. aureus* clinical isolates carry one or more prophages, and prophage content diverges markedly between community-acquired and healthcare-associated lineages (HA-MRSA), contributing to their distinct clinical presentations [11,12]. Yet these elements have historically been examined through a purely phage biological lens, leaving their dual significance as virulence amplifiers and as rich repositories of therapeutic molecules underappreciated. The phageome, defined as the population of bacteriophages within a given environment, is recognized as a significant factor that regulates the microbiome. Even though the association between skin and bacterial microbiome has been widely examined, the role of the phageome in skin remains insufficiently explored [13].

The recognition that prophages do not merely hitchhike on pathogen genomes. Instead, actively remodeling host physiology, immunity, and competitive fitness has crystallized a new conceptual framework [10,14]. In parallel, the antibiotic resistance crisis has catalyzed renewed interest in phage-derived antimicrobials as alternatives or adjuncts to conventional therapy. Prophage-encoded enzymes, endolysins, holins, and polysaccharide depolymerases have demonstrated efficacy against biofilm-forming, antibiotic-resistant skin pathogens in preclinical models, and the first clinical-stage candidates are advancing through trials [15,16]. This review examines the mechanistic duality of prophages in the context of skin infection as molecular architects of pathogen virulence and as a reservoir of precision therapeutics. We argue that integrating both perspectives is essential for harnessing prophage biology as a next-generation strategy for skin infection management.

## 2. Prophage-Driven Virulence: Mechanisms and Clinical Consequences

### 2.1. Toxin Genes and Lysogenic Conversion

**Figure 1:**
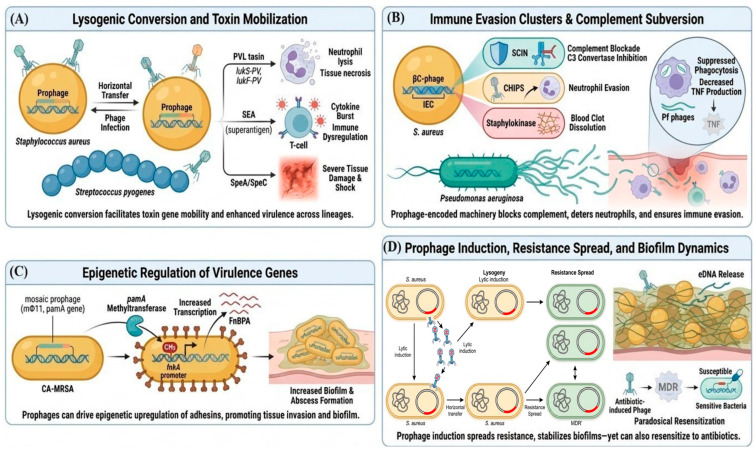
Prophage elements contribute to bacterial virulence by (**A**) the mobilization of toxin genes and lysogenic conversion, (**B**) prophage-encoded immune evasion cluster, (**C**) epigenetic regulation of virulence genes, and (**D**) stress-induced prophage activation, resistance dissemination, and eDNA release.

Various prophage elements, their virulence mechanisms, and clinical significance in skin pathogens are summarized in Table 1. The clearest manifestation of prophage-driven pathogenesis is the mobilization of toxin genes through lysogenic conversion (Figure 1A). Panton–Valentine leukocidin (PVL), encoded by lukS-PV and lukF-PV on lysogenic phages related to φSLT, is a bicomponent pore-forming toxin that mediates leukocyte lysis and tissue necrosis [17]. Its prophage-borne nature enables horizontal transfer between strains, explaining in part the epidemiological success of community-associated methicillin-resistant *S. aureus* (CA-MRSA) lineages such as USA300 in causing recurrent skin abscesses and necrotizing fasciitis [18]. Staphylococcal enterotoxin A (SEA), a superantigen that contributes to immune dysregulation in skin diseases, is encoded near the attachment sites of multiple staphylococcal prophages, enabling their efficient spread across lineages through lysogenic conversion [19]. In *S. pyogenes*, the pyrogenic exotoxins SpeA and SpeC superantigens causally linked to streptococcal toxic shock syndrome and necrotizing fasciitis are similarly prophage-borne. SpeA was among the first identified prophage-encoded virulence factors, residing on phage T12, and SpeC on related elements. Prophage carriage of these superantigens accounts for significant strain-level variability in *S. pyogenes* disease severity [20].

### 2.2. Immune Evasion Clusters and Complement Subversion

Beyond direct cytotoxicity, prophages encode sophisticated immune evasion machinery. The immune evasion cluster (IEC) of *S. aureus*, residing on β-haemolysin-converting bacteriophages (βC-φs), encodes the staphylococcal complement inhibitor (SCIN), the chemotaxis inhibitory protein of *S. aureus* (CHIPS), and staphylokinase proteins that collectively disable complement activation, impair neutrophil recruitment, and dissolve fibrin to promote bacterial dissemination (Figure 1B) [10,21]. The IEC is absent from many environmental *S. aureus* strains and present primarily in human-adapted lineages, underscoring the role of βC-φ acquisition in the evolutionary transition to a skin-invasive pathogen [22,23]. In *P. aeruginosa*, the filamentous prophage Pf (Pf4 and related variants) represents an entirely distinct class of virulence-associated prophage element. Pf phage, produced at a detectable level by a subset of *P. aeruginosa* cells at infection sites, is internalized by innate immune cells and triggers a Toll-like receptor 3-TIR-domain-containing adapter-inducing interferon-β (TLR3-TRIF)-mediated antiviral response that suppresses phagocytosis and tumor necrosis factor (TNF) production, effectively protecting the bacterium from clearance [24,25]. In a cohort of chronically infected wound patients, Pf phage was detected in two-thirds of *P. aeruginosa*-positive wounds, correlating with treatment failure, implicating prophage-mediated immune subversion directly in clinical chronicity. This study included chronic wound infection in humans, mice, and Yorkshire/Landrace pigs (*n* = 36, *n* = 6, *n* = 8, respectively). Experimental control settings included phosphate-buffered saline (PBS) control, heat-killed PAO1, vehicle control, mock-treated cell cultures, and a standardized splinted wound model. The study demonstrated statistically significant differences in wound-healing outcomes, including impaired re-epithelialization in positive pf infection. It delayed wound closure, with reported p-values such as *p* < 0.0001, *p* = 0.013, and *p* = 0.033 [26].

### 2.3. Epigenetic Regulation of Virulence: The Methyltransferase Paradigm

A conceptually distinct mechanism of prophage-mediated virulence emerged recently from the molecular epidemiology of CA-MRSA. The acquisition of a mosaic prophage, mΦ11, carrying an adenine methyltransferase gene (*pamA*), was sufficient to increase skin abscess virulence in a murine model without encoding any known toxin or fitness determinant [27,28]. The *pamA* methyltransferase epigenetically upregulates the expression of fibronectin-binding protein A (FnBPA), an established adhesin and biofilm promoter, through DNA methylation-dependent transcriptional reprogramming. The inactivation of *fnbA* abolished the virulence-enhancing effect of *pamA*, confirming FnBPA as a *pamA*-specific downstream effector (Figure 1C) [27,29]. This finding establishes prophage-encoded epigenetic enzymes as a previously unrecognized class of virulence modulator acting not through direct cytotoxicity but through remodeling the host transcriptome. This mechanism is likely widespread: the extensive repertoire of methyltransferase-encoding prophages in *S. aureus* isolates suggests that epigenetic virulence regulation via prophage acquisition may have contributed to the emergence of multiple clinically dominant lineages.

### 2.4. Prophage Induction, Biofilm, and Resistance Dissemination

Prophage induction, the switch from lysogeny to active lytic replication, is triggered by the bacterial SOS response, which is activated by antibiotics (particularly fluoroquinolones and beta-lactams), UV radiation, and sublethal stress [30]. Antibiotic treatment may accelerate resistance spread. SOS-triggered induction liberates phage particles that can transduce resistance-encoding DNA segments to neighboring susceptible cells, both within and between species (Figure 1D) [31]. In biofilm-forming *S. aureus* communities colonizing wound and skin surfaces, prophage induction contributes to extracellular DNA (eDNA) release, which reinforces biofilm matrix architecture and elevates antimicrobial tolerance. The specific mechanism of eDNA in biofilm formation functions as a structural component that enhances adhesive strength and maintains structural stability. Experimental evidence showed that DNase treatment degrades eDNA, causing biofilm detachment and structural collapse. Moreover, quantitative analysis demonstrates that the wild-type UAMS-1 biofilm contains approximately five times more eDNA than the *cidA* mutants, underscoring its critical role in biofilm formation [32]. Nonetheless, this induction vulnerability carries therapeutic relevance, as phage-driven selective pressure in MRSA populations has been shown to modulate antibiotic resistance through alternative transcriptional programs [33].

**Table 1:** List of prophages in skin infection pathogens and their encoded virulence factors.

Pathogen	Prophage/ Element	Encoded Factor	Mechanism of Virulence	Clinical Significance in Skin Infections	References
*S. aureus* (CA-MRSA USA300)	mΦ11 (mosaic prophage)	*pamA* (adenine methyltransferase)	Epigenetic upregulation of FnBPA via DNA methylation promotes in vivo biofilm formation and adhesion	Increased skin abscess size and severity; drove outbreak dissemination of CA-MRSA USA300-BKV variant	[27]
*S. aureus*	φSLT and related PVL phages	Panton–Valentine leukocidin (LukS-PV, LukF-PV)	Bicomponent pore-forming cytotoxin; lyses leukocytes and induces tissue necrosis	Furunculosis, skin abscesses, necrotizing fasciitis; a marker of CA-MRSA virulence	[18]
*S. aureus*	β-haemolysin-converting phages (βC-φs)	IEC (SCIN, CHIPS, staphylokinase, SEA)	Complement inhibition, neutrophil chemotaxis blockade, fibrin dissolution, superantigen-mediated T-cell activation	Immune escape in skin/soft tissue infections; elevated disease severity in chronic skin conditions	[10,21]
*S. aureus*	φETA and related phages	Exfoliative toxins A and B (ETA, ETB)	Serine protease activity targeting desmoglein-1 disrupts epidermal intercellular adhesion, causing superficial blistering without bacterial invasion of deeper tissue	Staphylococcal scalded skin syndrome (SSSS) and bullous impetigo; predominantly affects neonates and immunocompromised patients; ETA encoded on prophage, ETB on plasmid in most strains	[34]
*S. aureus*	SaPI1 (Staphylococcal pathogenicity island; phage-mobilized element)	Toxic shock syndrome toxin-1 (TSST-1)	Superantigen; binds MHC class II outside peptide-binding groove; activates up to 20% of T-cell pool, causing massive cytokine storm and systemic vasodilation	Staphylococcal toxic shock syndrome presenting with diffuse macular erythroderma and skin desquamation; SaPI1 is mobilized and transferred by helper phages, driving inter-strain spread	[35]
*S. pyogenes*	Phage T12 and related elements	Streptococcal pyrogenic exotoxins SpeA, SpeC	Superantigen activity; massive polyclonal T-cell activation; systemic cytokine storm	Streptococcal toxic shock syndrome, necrotizing fasciitis, scarlet fever; strain-level virulence variability	[20]
*S. pyogenes*	Prophage-encoded DNases (Sda1/SpnA)	Streptococcal DNase Sda1; SpnA streptodornase	Degradation of neutrophil extracellular traps (NETs) prevents NET-mediated bacterial killing, enabling bacteremia and systemic spread from the initial skin entry site	Essential for invasive progression from superficial skin infection to necrotizing fasciitis and bacteremia; elevated Sda1 carriage in invasive M1T1 lineages	[36]
*S. pyogenes*	Phage-encoded hyaluronidase locus (hylP)	Phage hyaluronidase HylP	Depolymerizes hyaluronic acid capsule and host connective tissue extracellular matrix; facilitates bacterial dissemination through dermis and subcutaneous tissue	Promotes spread of streptococcal cellulitis and impetigo beyond the primary infection site; higher hyaluronidase activity correlates with increased invasiveness in clinical *S. pyogenes* isolates	[37]
*P. aeruginosa*	Pf phage (Pf4; filamentous prophage)	Immunomodulatory phage coat proteins	Internalization by host immune cells; TLR3-TRIF-mediated antiviral response suppresses phagocytosis and TNF; promotes biofilm formation	Chronic wound infection persistence; Pf detected in ~65% of Pa-positive non-healing wounds; correlates with treatment failure	[24]

## 3. Prophage-Derived Molecules as Antimicrobials

### 3.1. Endolysins: The Frontline Therapeutic Class

**Figure 2:**
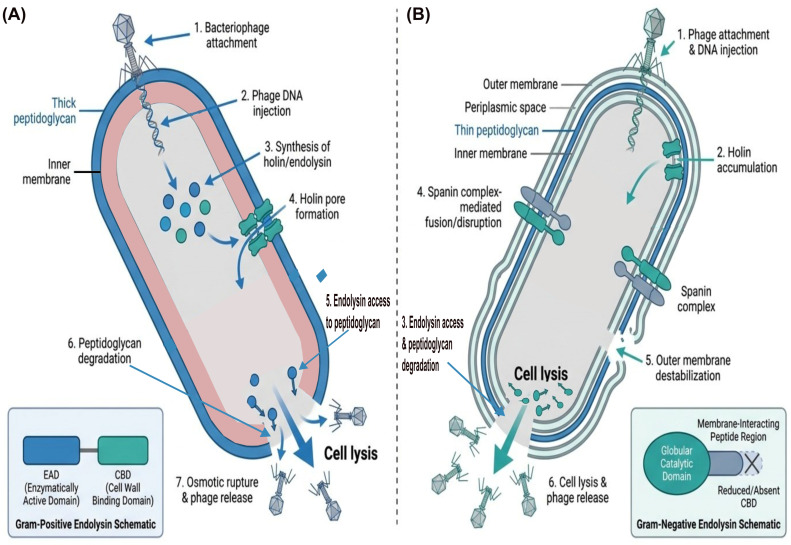
Natural mechanism of endogenous endolysins during phage-mediated lysis in both Gram-positive and Gram-negative bacteria. (**A**) The phage-mediated mechanism of cell lysis in Gram-positive bacteria employs holins and endolysins to degrade the peptidoglycan layer, allowing for the release of new phages. (**B**) The phage-mediated mechanism of cell lysis in Gram-negative bacteria involves holins, endolysins, and spanin systems that disrupt the bacterial cell envelope and facilitate phage release.

**Figure 3:**
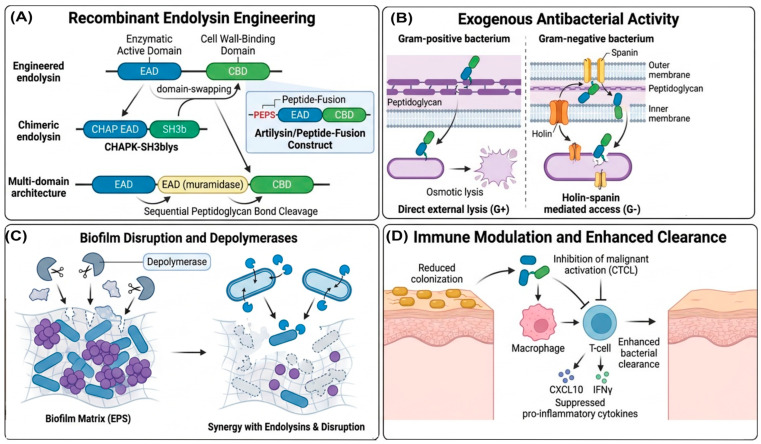
Engineered and therapeutic application of recombinant endolysin. (**A**) Engineered strategies for recombinant endolysin, including the development of engineered chimeric lysin and artilysins. (**B**) Exogenous application of endolysin showed direct antibacterial activity against Gram-positive bacteria, while the Gram-negative bacteria require disruption of the outer membrane. (**C**) Depolymerase disrupts the biofilm matrices, thereby enhancing endolysin efficacy through synergistic interactions. (**D**) Immune modulation and enhanced bacterial clearance, followed by endolysin therapy.

Based on mechanistic insight into prophage in pathogenesis, this section examines how the prophage-derived molecule is engineered into therapeutic strategies against skin pathogens. Endolysins are phage-encoded peptidoglycan hydrolases that degrade the bacterial cell wall in the terminal stage of the lytic cycle through natural holin-mediated membrane permeabilization in both Gram-positive and Gram-negative bacteria, with Gram-negative bacteria requiring additional spanin-mediated outer membrane disruption for complete lysis (Figure 2A,B). The therapeutic potential of prophage-derived molecules against skin pathogens was outlined in Table 2. Applied exogenously to Gram-positive pathogens, whose peptidoglycan layer is directly accessible from the outside, endolysins induce rapid osmotic lysis without requiring cellular uptake or holin partners [38]. Their modular architecture, with an N-terminal enzymatically active domain (EAD) and a C-terminal cell wall-binding domain (CBD), provides a rational basis for engineering, including domain-swapping to expand host range and enhance catalytic efficiency (Figure 3A) [38]. Against *S. aureus* and MRSA, multiple endolysin candidates have progressed from bench to clinical evaluation. Staphefekt SA.100, formulated as a topical gel, showed efficacy against diverse *S. aureus* strains in early skin infection studies [39]. Its engineered successor, XZ.700, outperformed SA.100 in head-to-head comparisons, selectively killing *S. aureus* without inducing resistance, and eliminated the pathogen on human skin explant models when formulated as cream or gel [16]. An ex vivo study demonstrated that XZ.700 not only cleared cutaneous *S. aureus* colonization but also suppressed inducible cytokine production and blocked *S. aureus*-driven malignant T-cell activation in cutaneous T-cell lymphoma (CTCL), extending its therapeutic relevance beyond simple bacterial killing to immune modulation in malignant skin disease. They used three independent healthy skin samples. Experimental controls included a tryptic soy broth control, a patient-derived *S. aureus* supernatant, and mock-treated cultures with or without XZ.700. Cytokine and chemokine expression analysis revealed that XZ.700 effectively blocked the CXCL10 and IFNγ induction. At the same time, IL-6 and IL-37 levels remained unchanged (Figure 3D). Statistical analysis was performed using one-way ANOVA with the Dunnett multiple comparison test and Student’s t-test, with significance thresholds of *p* < 0.05, *p* < 0.001, and *p* < 0.0001 [40]. The endolysin SAL200, engineered from the staphylococcal phage SAL-1 with a CHAP-amidase architecture, completed the Phase I clinical trials, demonstrating favorable pharmacokinetics and tolerability following intravenous administration in healthy volunteers, an important milestone for this therapeutic class [41]. Discovery-focused work has also tapped the skin microbiome prophageome: mining single-cell genomes from skin swab samples identified 96 endolysin genes from commensal *Staphylococcus* species, of which *in silico* peptide fragmentation yielded 37 novel antimicrobial peptides with predicted activity against antibiotic-resistant pathogens, antifungal properties, and molecular docking affinity for key therapeutic targets, including *S. epidermidis* autolysin and the beta-lactamase VIM-2 [42]. These findings establish the commensal skin phageome as a structurally diverse and clinically relevant source of next-generation antimicrobial peptides.

### 3.2. Holins, Spanins, and Depolymerases: An Underexplored Arsenal

In addition to endolysin, prophage-derived proteins, which were originally evolved for bacterial lysis, can be repurposed as therapeutic agents and represent a largely untapped frontier in antimicrobial research. Holins are small, hydrophobic membrane proteins that perforate the cytoplasmic membrane at a precisely timed point in the lytic cycle, controlling endolysin access to peptidoglycan [43,44]. When applied exogenously, specific holins such as Hol-4086 have demonstrated direct bactericidal activity against *S. aureus* and *Enterococcus faecalis* and, when co-expressed or combined with endolysins, show a broader lytic range that extends to some Gram-negative pathogens. Spanins, phage proteins that disrupt the Gram-negative outer membrane during the final lysis step, are attracting attention as potential therapeutic agents against MDR wound pathogens such as *P. aeruginosa* and *Acinetobacter baumannii*, for which endolysins alone are insufficient due to the outer membrane barrier (Figure 3B) [45,46,47,48]. Polysaccharide depolymerases are phage-encoded enzymes that degrade capsular polysaccharides and biofilm exopolysaccharide matrices, synergizing with endolysins by dismantling the protective extracellular matrix that otherwise limits lysin access to the peptidoglycan layer (Figure 3C) [49]. Unlike conventional antibiotics, depolymerases specifically target the polysaccharide component and do not depend on phage-mediated bacterial lysis, effectively diffusing through biofilm, weakening biofilm architecture, and increasing antimicrobial susceptibility. Their therapeutic potential is influenced by the infection context and enhanced when used in combination with antimicrobial treatments [50]. Recombinant depolymerases effectively inhibit *A. baumannii* biofilm in vitro, reduce biofilm biomass, and weaken structural integrity. In combination with colistin, it further enhances bacterial clearance. Capsule degradation exposes bacteria to complement activation and phagocytosis, increasing serum antimicrobial activity [51]. These multi-component combinations, which recapitulate elements of the natural phage lytic machinery, are increasingly viewed as necessary for addressing biofilm-associated skin and wound infections refractory to single-agent approaches and have demonstrated enhanced bacterial clearance in preclinical wound models [26].

**Table 2:** Prophage-derived molecules with therapeutic potential against skin infection pathogens.

Molecule	Class	Origin	Target Pathogen	Clinical Findings	Antimicrobial Activity	Stage	References
XZ.700	Recombinant endolysin	Staphylococcal phage	*S. aureus*, MRSA	Eliminates *S. aureus* on human skin explant model (cream/gel); suppresses cytokine production; blocks malignant T-cell activation in CTCL ex vivo	>4 log10 reduction in CFU at ≥1 µg/mL	Preclinical/early clinical	[40]
Staphefekt SA.100	Recombinant endolysin	Staphylococcal phage	*S. aureus*	Demonstrated skin infection activity as a topical formulation; selective staphylococcal killing; well-tolerated in early evaluation	ND	Clinical evaluation	[16]
SAL200	Endolysin (CHAP-amidase)	Phage SAL-1	*S. aureus*, MRSA	Phase I: IV administration in healthy volunteers; favorable PK and tolerability; rapid bactericidal activity; no reported resistance induction	0.078 µg/mL (MBC)	Phase I completed	[41]
CHAPK-SH3blys	Chimeric endolysin	Domain-swapped staphylococcal phage lysins	*S. aureus*, MRSA	Biocompatible with human cell lines; no resistance after prolonged sub-MIC exposure	3.9 µg/mL (MIC)	Preclinical	[15]
Hol-4086	Holin	Staphylococcal phage	*S. aureus*, *E. faecalis*	Direct bactericidal membrane disruption; broader host range than endolysins alone; synergistic with endolysins against polymicrobial targets	ND	Preclinical	[45]
Skin phageome-derived AMPs (37 novel peptides)	Antimicrobial peptides (AMP)	Human skin microbiome prophages	*S. epidermidis*, MDR pathogens	In silico screening of 96 endolysin genes from skin commensal staphylococci; 7 peptides with structural stability in MD simulations; docking activity vs autolysin and VIM-2 beta-lactamase; predicted antifungal and antiviral properties	ND	Discovery phase	[42]
LysK	Endolysin (CHAP-amidase-SH3b, three-domain)	Staphylococcal phage φK	*S. aureus,* MRSA, coagulase-negative *staphylococci*	Prototype tri-domain anti-staphylococcal endolysin; rapid lysis of MRSA at low concentrations; active against diverse clinical MRSA isolates, including livestock-associated strains; synergistic killing with lysostaphin; founding scaffold for most chimeric lysin designs, including CHAPK-SH3blys	99% CFU reduction within 1 h (500 μL)	Preclinical	[52]
PlySs2	Endolysin (CHAP-SH3b)	Streptococcal prophage	*S. pyogenes*, *S. aureus*, MRSA	Broad Gram-positive spectrum; active against *S. pyogenes* and MRSA at low concentrations; efficacy in murine bacteremia model; protease-resistant at physiological pH; maintains activity in wound fluid; candidate for skin and wound infection	MIC: *S. pyogenes*: 128–256 µg/mL MRSA: 16–32 µg/mL, *S. aureus*: 16 µg/mL.	Preclinical	[53]

ND: Not determined.

## 4. Engineering Prophage Biology for Precision Skin Therapeutics

### 4.1. Chimeric Lysins and Host Range Expansion

Domain engineering strategies have substantially expanded the therapeutic utility of endolysins beyond their natural host ranges. By combining EADs and CBDs from phylogenetically distinct endolysins, so-called chimeric approaches, researchers have generated constructs that target multiple species, circumvent resistance to parental enzymes, and display synergistic killing when combined [38]. A chimeric endolysin approach has produced CHAPK-SH3blys, combining the CHAP domain of CHAPK with the SH3b cell wall-binding domain of lysostaphin. This construct demonstrated a minimum inhibitory concentration (MIC) of 3.9 µg/mL against diverse *S. aureus* strains implicated in chronic wound infections and produced up to a 4-log reduction in viable biofilm cells under dynamic wound-like conditions, without measurable resistance induction after prolonged sub-MIC exposure [15,54]. Multi-domain architectures that include both an endopeptidase and a muramidase EAD have further demonstrated intramolecular synergy, exploiting the sequential cleavage of distinct peptidoglycan bonds to enhance bactericidal potency and reduce the mutational pathways available for resistance. The rate of resistance emergence against chimeric endolysins in *S. aureus* appears significantly lower than against antibiotics, an observation with critical translational implications for skin infections caused by strains already resistant to multiple drug classes [38,55,56].

### 4.2. CRISPR-Encoded Prophage Delivery Systems

A conceptually distinct engineering strategy couples temperate phage delivery with Clustered Regularly Interspaced Short Palindromic Repeats (CRISPR)-Cas antimicrobial payloads. Phage-delivered Cas9 constructs can be programmed to cleave chromosomal sequences, resistance genes, virulence loci, or pathogen-specific sequence signatures within the target organism, inducing lethal genomic damage with species-level precision [57]. In *S. aureus,* a temperate phage-based delivery system carrying CRISPR-Cas9 directed against *mecA,* the resistance determinant encoding penicillin-binding protein 2a, sensitized MRSA to beta-lactam antibiotics, and killed target strains with sequence specificity that spared commensal staphylococci [58,59,60]. This approach is particularly compelling in skin infection, where preservation of commensal flora is clinically important, unlike broad-spectrum antibiotics. Phage-delivered CRISPR constructs can be designed to target only the pathogenic lineage without disrupting the commensal microbiome that provides colonization resistance against re-infection. Further engineering of these constructs to encode multiple guide RNAs targeting distinct chromosomal loci may reduce the probability of resistance emergence and expand coverage to heteroresistant populations.

### 4.3. Mining the Skin Phageome and Topical Delivery Challenges

The human skin harbors a compositionally stable virome dominated by prophages of commensal *Staphylococcus*, *Cutibacterium*, and *Corynebacterium* species, forming what has been called the skin phageome. This reservoir has evolved in intimate co-adaptation with both human skin physiology and skin-associated pathogens, making it an intrinsically relevant source of antimicrobial molecules for topical applications [42,61]. Systematic computational and experimental mining approaches, including single-cell bacterial genomics, metagenomic phage discovery, and antimicrobial peptide prediction platforms, are increasingly being applied to identify prophage-derived antimicrobial candidates adapted to the skin microenvironment [62,63]. However, the identification of prophage-derived molecules alone is insufficient for clinical translation because their efficacy also depends on efficient delivery and stability in the wound environment. Their large molecular weight, susceptibility to protease degradation in wound exudate, and limited penetration of intact stratum corneum require formulation innovation. Therefore, nanotechnology-based delivery vehicles, including liposomes, polymeric nanoparticles, and biocompatible hydrogel matrices, have demonstrated improved endolysin stability and controlled release at skin infection sites and represent a necessary adjunct to molecule discovery for topical therapeutic development [49,64].

## 5. Conclusions and Future Perspectives

Prophages occupy a paradoxical position in skin pathogen biology. The most potent contributors to bacterial virulence and resistance simultaneously encode a structurally and mechanistically rich arsenal of molecules with transformative therapeutic potential. The conceptual framework emerging from recent work is one of transforming bacterial strategies into therapeutic tools, directing the molecular tools that prophages have evolutionarily refined against pathogens, rather than permitting them to amplify pathogen fitness. The discovery that prophage-encoded epigenetic enzymes such as *pamA* can reshape pathogen virulence through DNA methylation-dependent transcriptional reprogramming [27] represents a paradigm shift that is likely to reveal further epigenetic virulence mechanisms in other prophage-harboring skin pathogens.

Several areas demand prioritized investigation. First, the dual-use risk of prophage induction as a therapeutic strategy and, specifically, the concurrent release of virulence-encoding phage particles alongside the intended bactericidal response, requires systematic assessment in clinically relevant skin infection models before this approach can be safely translated. Second, the skin phageome of geographically and clinically diverse human populations remains largely uncharacterized. Sampling commensal prophage diversity beyond the Western, industrialized settings that dominate current datasets is expected to uncover antimicrobial molecules active against pathogens beyond the *S. aureus*/MRSA-centric paradigm. Third, regulatory frameworks for prophage-derived protein therapeutics, though advancing, remain less mature than those governing antibiotic drugs: accelerated regulatory science engagement is essential for this class to benefit patients with treatment-refractory skin infections.

The most productive near-term frontiers are likely to be combination strategies: endolysins with depolymerases to address biofilm-protected skin pathogens, prophage induction to resensitize MDR strains to existing antibiotics, and CRISPR-phage conjugates to eliminate resistance determinants with species-level precision. Prophage silencing maintains lysogenic dormancy by repressing prophage gene expression. For example, in *P. aeruginosa*, the kinase–kinase–phosphatase (KKP) module controls prophage activation by altering the phosphorylation of MvaU, a host nucleoid-associated protein that acts as a prophage silencer [65]. The lysogenic control mechanism serves as a therapeutic approach against bacterial persistence and chronic infection. The possibility of prophage silencing targeting the transcriptional regulators that maintain lysogeny to prevent virulence gene expression adds a further dimension that remains essentially unexplored. Prophages in skin pathogens are neither silent passengers nor unidirectional villains: they are dynamic regulatory elements whose clinical significance and therapeutic promise are only beginning to be fully appreciated.

## Data Availability

No new data were created or analyzed in this study.

## Abbreviations

The following abbreviations are used in this manuscript:

AMP	Antimicrobial peptide
βC-φ	Beta-haemolysin-converting bacteriophage
CA-MRSA	Community-associated methicillin-resistant *Staphylococcus aureus*
CBD	Cell wall-binding domain
CFU	Colony-forming unit
CHIPS	Chemotaxis inhibitory protein of *S. aureus*
CTCL	Cutaneous T-cell lymphoma
CRISPR	Clustered Regularly Interspaced Short Palindromic Repeats
EAD	Enzymatically active domain
eDNA	Extracellular DNA
ETA	Exfoliative toxin A
ETB	Exfoliative toxin B
FnBPA	Fibronectin-binding protein A
HA-MRSA	Healthcare-associated methicillin-resistant *Staphylococcus aureus*
IEC	Immune evasion cluster
KKP	Kinase–kinase–phosphatase
MDR	Multidrug resistance
MBC	Minimum bactericidal concentration
MIC	Minimum inhibitory concentration
PBS	Phosphate-buffered saline
PVL	Panton–Valentine leukocidin
SEA	Staphylococcal enterotoxin A
SCIN	Staphylococcal complement inhibitor
SSSS	Staphylococcal scalded skin syndrome
TLR3	Toll-like receptor 3
TRIF	TIR-domain-containing adapter-inducing interferon-β
TNF	Tumor necrosis factor
TSST-1	Toxic shock syndrome toxin-1
